# Development of Metronidazole Loaded Chitosan Nanoparticles Using QbD Approach—A Novel and Potential Antibacterial Formulation

**DOI:** 10.3390/pharmaceutics12100920

**Published:** 2020-09-25

**Authors:** Nagaraja Sreeharsha, Kuldeep Rajpoot, Muktika Tekade, Dnyaneshwar Kalyane, Anroop B. Nair, Katharigatta N. Venugopala, Rakesh K. Tekade

**Affiliations:** 1 Department of Pharmaceutical Sciences, College of Clinical Pharmacy, King Faisal University, Al-Ahsa 31982, Saudi Arabia; anair@kfu.edu.sa (A.B.N.); kvenugopala@kfu.edu.sa (K.N.V.); 2 Department of Pharmaceutics, Vidya Siri College of Pharmacy, Off Sarjapura Road, Bangalore 560035, India; 3 Pharmaceutical Research Project Laboratory, Institute of Pharmaceutical Sciences, Guru Ghasidas Vishwavidyalaya (A Central University), Bilaspur 495009, India; kuldeep_sagar06@rediffmail.com; 4 TIT College of Pharmacy, Technocrats Institute of Technology Campus, Anand Nagar, Raisen Road, Bhopal 462021, India; muktikarakeshtekade@gmail.com; 5 National Institute of Pharmaceutical Education and Research (NIPER)–Ahmedabad, Opposite Air Force Station Palaj, Gandhinagar 382355, India; dkalyane@gmail.com (D.K.); rakeshtekade@gmail.com (R.K.T.); 6 Department of Biotechnology and Food Technology, Durban University of Technology, Durban 4001, South Africa; 7 Department of Pharmaceutical Technology, School of Pharmacy, The International Medical University, Kuala Lumpur 57000, Malaysia; 8 Department of Materials Engineering, Indian Institute of Technology-Jammu, Jagti, PO Nagrota, Jammu 181 221, India

**Keywords:** metronidazole, nanoparticles, chitosan, QbD, Eudragit S100, colon-specific delivery

## Abstract

The aim of this study was to design, optimize, and develop metronidazole (Met) loaded nanoparticles (MetNp) by employing quality-based design (QbD) as well as a risk assessment methodology. A fractional factorial design was used by selecting five independent variables viz., chitosan concentration, tripolyphosphate concentration, and acetic acid concentration as material attributes, stirring speed, and stirring time as process parameters, whereby their influence on two dependent variables such as particle size (PS) and %entrapment efficiency (%EE) was studied. MetNp were synthesized by employing an ionic-gelation technique and optimized formula obtained from the QbD design study. PS and %EE studies revealed the formation of MetNp with 558.06 ± 2.52 nm and 59.07 ± 2.15%, respectively. Furthermore, a Met release study in various simulated gastro-intestinal media suggested pH-triggered (pH > 7.0) and sustained release profile of Met from Eudragit S100 enteric-coated MetNp capsule (MetNp cap). Moreover, the stability investigation of formulations confirmed good stability with respect to their PS and residual drug content (RDC) at different temperature conditions. In conclusion, the QbD method was effectively utilized in the development of MetNp and enteric-coated MetNp cap depicting their potential to release Met through MetNp cap only in the colon region and can be utilized for the treatment of amoebiasis in the colon.

## 1. Introduction

Entamoeba (E.) histolytica is a protozoan parasite, which exists as a single-cell. It is responsible for instigating amoebiasis in the large intestine. Trophozoites of E. histolytica may enter the colonic epithelium resulting in amoebic colitis [1]. For the treatment of amoebiasis in the intestine, two drugs, namely metronidazole (Met) [2] and tinidazole [3], are widely used. To attain maximum effect against E. histolytica, these drugs should be delivered selectively in the colon region where the trophozoites live and grow [4].

**Figure 1:**
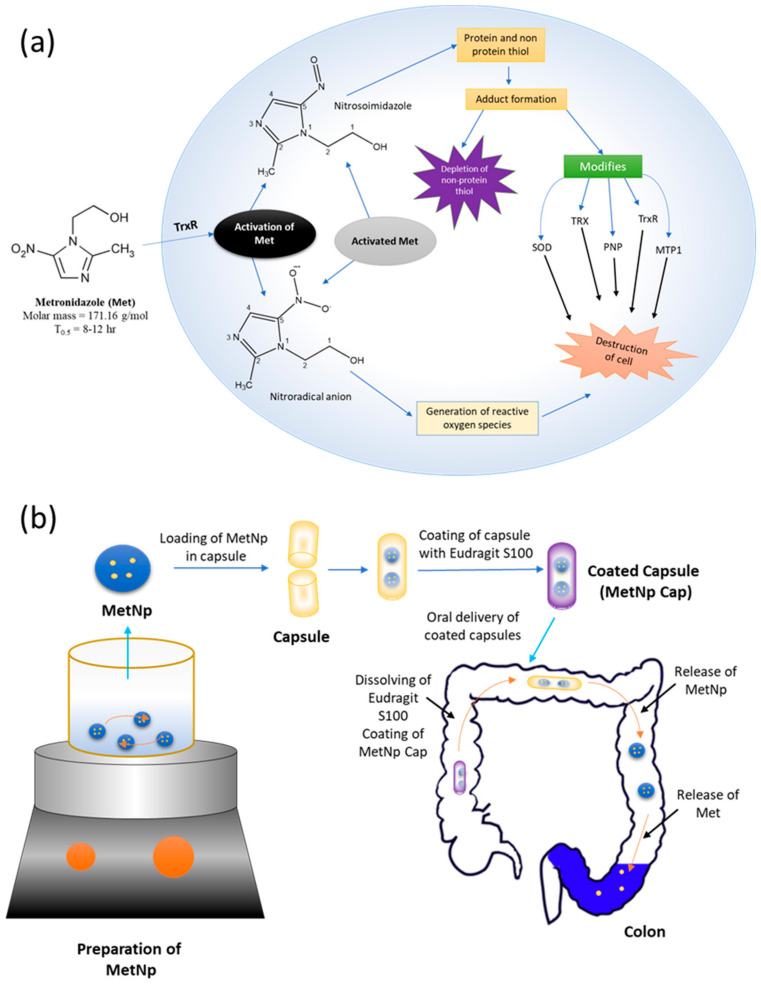
(**a**) Chemical structure and mechanism of action of Met. (**b**) Schematic showing preparation of MetNp and its delivery to the colon for treatment of amoebiasis.

Met is the preferred antibiotic for the treatment of intestinal amoebiasis (Figure 1a) [5]. Importantly, Met showed activity specifically toward anaerobes [2]. The mechanism of the treatment is not fully understood, but involves the inhibition of DNA synthesis and the breaking of DNA into a single-strand by the oxidation, which is generally responsible for the killing of cells [6]. However, Met is not well suited to oral delivery, as it is absorbed completely within a small-time and obtains peak plasma concentrations after 1–2 h [6].

In traditional approaches, where the drug is delivered through the oral route, drug molecules usually dissolve and are absorbed through the gastrointestinal (GI) tract [7]. Available approaches for the treatment of amoebiasis via the oral route face some problems including: degradation of the drug due to acidic pH in the stomach and presence of various enzymes; rapid (1 h) peak plasma concentration; and nonspecific and inefficient delivery of the drug. [7]. Many strategies have been employed for targeting drugs in the colon milieu including: (a) a layer of pH-dependent polymer(s) that dissolve at pH equal to or higher than 7; (b) design of pulsatile and sustained release dosage forms [8]; and (c) the application of materials that might be digested entirely by the colonic bacteria [9].

Selected nanoparticles (NPs) have some essential characteristics that suggest their potential application to attain efficient targeting of the colon for the optimal effect of Met. These characteristics include: slight burst release of drug in the early phase; easy permeability; resistance to degradation; hydrophobicity; high surface charge; high drug loading; nontoxic nature; high binding as well as deep permeating ability through mucous membrane owing to their nanosize [10,11,12,13]. Several polymers such as alginate and chitosan (CH) [14] have been exploited not only for their mucoadhesive effect in GI but also for discovering their probable effectiveness in the colon milieu.

CH is a cationic polysaccharide, which rapidly affixes to mucous membrane with a negative surface charge [14]. It is a biodegradable polymer and exhibits minimum toxicity, which make it an ideal carrier for the delivery of several drugs [15]. It has been widely used as one of the components in the formulation of novel carrier systems such as NPs [16,17,18], microspheres [19], and microcapsules [20] in the treatment of various illnesses, including infections [21] and cancers [15]. After oral delivery, CH remains intact in both the stomach and the small intestine. However, when it reaches the colon region, it is selectively digested by the enzymes e.g., polysaccharidases [9]. Coating with polymers that are insoluble in aqueous medium offers a shielding effect for the drug in the superior region of the GI tract, and the drug is released in the colon, allowing subsequent digestion by the enzymes. Furthermore, coating with Eudragit S100 polymer guards the NPs against the acidic surroundings in the GI tract and thus allows the release of active molecules at the site of interest [7].

Following oral delivery, most of the drug is degraded by both GIT enzymes as well as acidic conditions in the stomach, and hence, very little drug reaches the target site and effectiveness is reduced. In addition, drug released in the stomach causes drug-associated toxicities, which leads to the noncompliance of patients. In order to maintain a therapeutic level of drugs, a high dose of the drug is required, and this further increases the cost of the drug and treatment [22]. To overcome these issues and achieve efficient results, an oral drug delivery system should be developed that has the potential to not only protect the drug in the gastric condition but also deliver a significant proportion of the drug to the target site.

**Figure 2:**
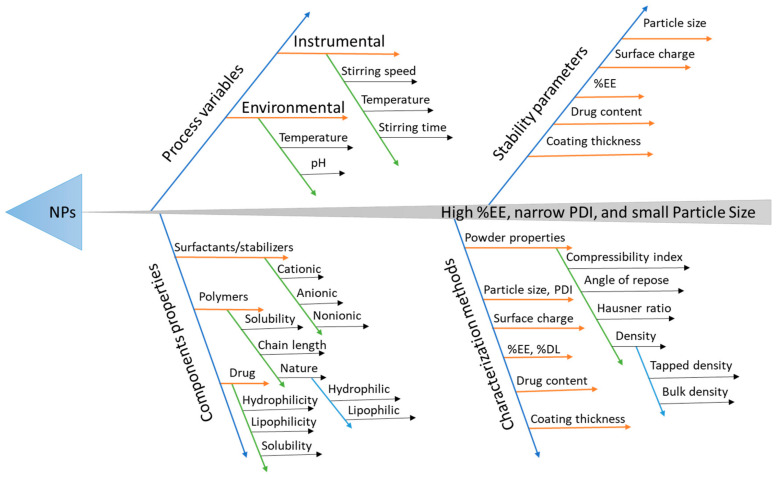
Ishikawa diagram depicting critical parameters that are considered in the development of nanoparticles (NPs).

At the present time, the interest in quality by design (QbD) is rapidly increasing for the systematic application of quality products to attain some specific goals—for instance, reduce the costs of product, and decrease the time to reach market, logical controls, and increased efficiency. It can also help to understand the important methods and product constraints that rely on risk measurement [23]. QbD has been widely employed and found to be beneficial to optimize various processes developing a more efficient and stable formulation (Figure 2) [24]. This study was aimed at designing and developing NP incorporating Met for the successful administration of bioactivity in the colon for treating amoebiasis (Figure 1b).

QbD assisted Met containing NPs (MetNp) were then analyzed for bulk and tapped density, particle size (PS), Hausner ratio, percent entrapment efficiency (%EE), angle of repose, and compressibility index. Afterward, the MetNp were loaded into a capsule coated with Eudragit S100 for colon-specific delivery and assessed for drug content, lock length, variation in the weight, Met release (in vitro), and stability.

## 2. Materials and Methods

### 2.1. Materials

Chitosan (CH) (viscosity grade 200–400 mPas, MW = 190–310 kDa), tripolyphosphate (TPP), Eudragit^®^ S100 (an enteric polymer), and FITC were procured from Sigma-Aldrich, St. Louis, MO, USA. Dialysis semipermeable membrane (MWCO, 10–12 KDa) and trehalose dihydrate were bought from HiMedia, Mumbai, India. All other excipients, as well as solvents utilized in the experiment, were of analytical grade.

### 2.2. Risk Identification

Different methods are used for the identification of risk factors that affects critical quality attributes (CQA). In this study, we used fishbone or Ishikawa or a cause and effect analysis diagram for the identification of the risk factors. Critical process parameters (CPP) and critical material attributes (CMA) were further screened out using the 2-level fractional factorial design (FFD).

### 2.3. Screening of Significant/Potential Risk Factors

To screen out CMA and CPP that affect product CQAs, we employed 2-level FFD. In this, we selected 5 independent variables as CPP based on the preliminary experiment and literature review: the concentration of CH, TPP, and acetic acid as material attributes; stirring speed; and stirring time. As it is a 2-level design, we selected a high (+1) and low (−1) level for each independent variable to estimate the effect it had on CQA. The low and high levels for each independent variable are shown in Table 1. Design-Expert 7.1 software (Stat-Ease Inc., Minneapolis, MN, USA) was utilized throughout the experimental design. The software provided 16 runs for this experimental design which were performed. The CQAs of our investigation were PS and EE. The screening of CMA and CPP was carried out based on the half-normal plot and Pareto chart analysis.

### 2.4. Optimization of CH NPs (CH-NPs) Using Response Surface Methodology (RSM)

The optimization of selected independent variables has been conducted by employing RSM in the Box–Behnken design (BBD). After using 2-level FFD, we found that 3 independent variables significantly affect the PS and EE of CH-NPs. These 3 factors were CH concentration, TPP concentration, and stirring time. Hence, for further optimization of these 3 critical variables, response surface BBD was applied. The other 2 variables, i.e., stirring time and acetic acid concentration, were kept constant. In this design, the variables were set to 3 levels: high (+1), medium (0), and low (+1). Table 2 shows the coded and actual values for all 3 independent variables. The 17 experimental runs were performed, and the results obtained were further evaluated. A quadratic equation was obtained in the form given below:

(1) $$Y=\gamma_{0}+\gamma_{1}X_{1}+\gamma_{2}X_{2}+\gamma_{3}X_{3}+\gamma_{12}X_{1}X_{2}+\gamma_{13}X_{1}X_{3}+\gamma_{23}X_{2}X_{3}+\gamma_{11}X_{1}^{2}+\gamma_{22}X_{2}^{2}+\gamma_{33}X_{3}^{2}$$

where Y denotes the response; X_1_, X_2,_ and X_3_ are the independent variables; X_1_X_2_, X_1_X_3_, and X_2_X_3_ are the interaction terms; $X_{1}^{2}$, $X_{2}^{2}$, and $X_{3}^{2}$ indicate the quadratic effects of given variables; γ_0_ is constant arithmetic mean response; and γ_1_, γ_2_, γ_3_, γ_12_, γ_13_, γ_23_, γ_11_, γ_22_, and γ_33_ are estimated coefficients for the respected variables. One-way analysis of variance (ANOVA) was employed to determine the level of significance of the model for each variable. The Box–Cox plot (a diagnostic plot) was used to estimate whether power transform was required or not. The interaction plots and 3-D RSM were used to depict the influence of variables on the response. The overall analysis design space (overlay plot) was generated to optimize the formula. 

### 2.5. Optimization of Design as Well as Validation of the Model

Validation of the model and design space was carried out to ensure the quality of the design space generated. The validation was carried out by performing the confirmatory experiments throughout the design space.

### 2.6. Formulation of MetNp

MetNp were synthesized by employing an ionic-gelation technique with small modifications [25]. The MetNp were synthesized by exploiting the principle of electrostatic attraction amongst polyanionic TPP and the primary –NH_2_ (cationic) group of the CH. In detail, CH was solubilized in acetic acid (1% *w*/*v*) solution in different concentrations (0.5, 1.0, and 1.5% *w*/*v*). On the other hand, TPP was solubilized in the aqueous phase at different concentrations (0.4, 0.8, and 1.2% *w*/*v*). Following this, Met was homogeneously dispersed in the previously prepared TPP solution, and then it was mixed slowly to CH solution under continuous agitation at different rpm (500, 750, and 1000 rpm). Thus, synthesized MetNp were centrifuged at 8000 rpm for 0.5 h (Sorvall legert XRT, Thermo Scientific, Waltham, MA, USA). The supernatant phase was removed, and NPs were redistributed in the saline medium (PBS, pH 6.8). Then, this dispersion was ultrasonicated (150 W for 2 min) so that these developed NPs disaggregated to form a single NP. The optimized MetNp lots were added to a deionized water solution comprising mannitol (1% *w*/*v*) as a cryoprotectant, and subjected to lyophilization at −20 and −50 °C for two one-day periods separated by a one-day interval, under vacuum pressure at 0.001 mbar (FD8508, Ilshin Biobase, Maxwellstraat, The Netherlands). Finally, MetNp were stored in glass vials in the desiccator.

### 2.7. Determination of PS

The hydrodynamic PS of MetNp were analyzed after subjecting samples to dynamic light scattering in Zetasizer Nano ZS 90 (Malvern instruments, Royston, UK) analogous to an earlier study performed by our group [16,26,27,28]. Samples were diluted (10-times) with deionized water at 25 ± 0.5 °C and were sonicated before examination. All results were recorded in triplicate.

### 2.8. Determination of %EE

To determine %EE of the Met, MetNp were first digested for 30 min in a solution of acetic acid (2% *v*/*v*) by employing a probe sonicator (Misonix, Farmingdale, NY, USA). Afterward, the samples were subjected to centrifugation to develop pellets of NPs at 12,000 rpm for 0.5 h at 25 ± 0.5 °C [29,30]. The supernatant after centrifugation was analyzed for Met concentration in a UV-visible spectrophotometer (Shimadzu, Kyoto, Japan) at λ_max_ = 277 nm to determine the %EE of Met by using Equation (2),

(2) $$\%\mathrm{EE}=\;\frac{X_{1}-X_{2}}{X_{1}}\;\times 100$$

where

*X*_1_ = Amount of Met added (mg);
*X*_2_ = Amount of free Met after centrifugation (mg).

### 2.9. Preparation Of Eudragit Coated Capsule Containing MetNp (MetNp Cap)

Hard gelatin capsules (Capsule size #4; weight 38 ± 3 mg) bearing MetNp powder blend were layered by plunging in a solution of Eudragit S100 (12% *w*/*v*), which was developed by incorporating Eudragit S100 in an organic solvent that was prepared by adding 5% *v*/*v* triethyl citrate (a plasticizer) in a solution of acetone: methanol (1:1, *v*/*v*). The coated capsules were examined for the amount of coating, which depicted approximately 4.0 mg/cm^2^. The amount of coating was determined by using Equation (3) given below.

(3) $$\mathrm{Amount}\;\mathrm{of}\;\mathrm{coating}=\frac{W_{\mathrm{ec}}-{\;W}_{c}}{S_{c}}$$

where

W_ec_ = Weight of enteric-coated capsule (mg);
W_c_ = Weight of uncoated capsule (mg);
S_c_ = Surface area of the uncoated capsule (cm^2^).

### 2.10. Determination of Bulk (ρ_o_)/Tapped (ρ_t_) Density

To determine bulk/tapped volume, nearly 5.0 g of the NPs-blend was placed in a measuring cylinder with 25 mL capacity. The bulk volume of the NPs was recorded, and then it was tapped 100 times to obtain the tapped volume [31,32]. The *ρ_o_*, as well as *ρ_t_* of the blend, were determined by using Equations (4) and (5), respectively,

(4) $$Bulk\;Density\;\left(\rho_{o}\right)\;=\frac{Weight\;of\;powder}{Bulk\;volume\;of\;powder}$$

(5) $$Tapped\;Density\;\left(\rho_{t}\right)=\frac{Weight\;of\;powder}{Tapped\;volume\;of\;powder}$$

### 2.11. Angle of Repose

The frictional forces that occur in the powder of NPs can be determined by measuring the angle of repose. The angle of repose is the highest feasible angle of the surface of the pile relative to the horizontal plane. This indicates the flow properties of the powder, which are essential for the compression of powder. It was determined by employing an open cylinder technique [33]. Here, a cylinder with both open ends was selected, and then an exactly weighed amount of testing powder-blend (5.0 g) was added to the cylinder. The cylinder was raised, and the height of the formed pile and the radius of the base was recorded. The angle of repose (*θ*) was calculated by using Equation (6):

(6) $$\theta =\tan^{-1}\frac{h}{r}$$

Here, “*r*” denotes radius while “*h*” denotes height.

### 2.12. Compressibility Index

The flowability, as well as size of the pack of the powder, are generally evaluated by relating *ρ_o_* and *ρ_t_* and also via determining the rate of flow of powder. To measure the compressibility, nearly 5.0 g of the NPs-blend was taken in a measuring cylinder with 25 mL capacity. The bulk volume of the NPs was recorded, and then it was tapped 100 times to obtain the tapped volume [31]. The following formula (Equation (7)) was used to calculate the Compressibility index:

(7) $$Compressibility\;index\;\left(\%\right)=\frac{Tapped\;density}{Bulk\;density}\times 100$$

### 2.13. Hausner Ratio

In order to find the Hausner ratio, the NPs-blend was placed in a cylinder with a volume capacity of 25 mL. The bulk volume of the blend was determined, and then it was again subjected to 100 tapings [31] to obtain the tapped volume. The following formula was used to calculate the Hausner ratio:

(8) $$Hausner\;ratio\;=\;\frac{Tapped\;density}{Bulk\;density}$$

### 2.14. Drug Content Assay of Blend

To determine drug content, a blend sample (nearly 20 mg) was placed into a pestle mortar and triturated. The triturated sample was digested by adding it into a solution containing 5 mL each of acetone, 0.5 M acetic acid, and 0.5 M NaOH and leaving overnight at 10 °C. The pH of the solution was adjusted to be slightly acidic. PBS (pH 7.4) was added to the digested homogenate and subjected to centrifugation for 15 min at 3000 rpm. Finally, the supernatant was used to determine % assay by UV-visible spectrophotometry (UV3600, Shimadzu, Kyoto, Japan) at 277 nm [34] using the following Equation (9):

(9) $$\%\;Assay=\frac{N_{act}}{N_{the}}\times 100$$

where *N_act_* and *N_the_* are the actual and theoretical amount of Met in the blend, respectively.

### 2.15. Weight Variation Test

This study was conducted to confirm that each of the capsules contains the appropriate quantity of the drug. A total of 20 capsules were selected, and their weight was determined individually via using analytical weighing balance. The average weight of all 20 capsules was determined, and then it was compared with the weight of individual capsules.

### 2.16. In Vitro Drug Release Study

This parameter is considered vital for the in vitro evaluation of dosage forms to determine the sustained release profile of the prepared MetNp and MetNp Cap. In vitro release of Met from MetNp was carried out in the USP XIII dissolution test apparatus (Paddle type). The Eudragit S100 coated hard gelatin capsules filled with MetNp were placed inside the dialysis membrane (Sigma-Aldrich, St. Louis, MO, USA) and then dipped in the various simulated gastric, intestinal, as well as GI fluids (SGF/SIF/SGIF, 900 mL) of different pH (i.e., SGF, pH 1.2; SGIF, pH 4.5; SIF, pH 7.4; and simulated colonic fluid (SCF, pH 7.0)) as listed in Table 3. SGF was prepared by adding 12,000 U/L pepsin in an aqueous solution, and its pH was adjusted to pH 1.2 using 0.1 M HCl. SIF was prepared by mixing 8400 U/L lipase solution in phosphate buffer (pH 7.4). SCF was prepared by adding 6 g/L pectinase solution in phosphate buffer (pH 7.0) [35].

At the time of the study, the temperature conditions of the fluid were kept at 37 ± 1 °C and stirred at 200 rpm. Aliquots from the medium were taken out at predetermined periods, and a fresh volume of fluid of respective pH was replaced to sustain the volume of the medium. The samples were assessed following appropriate dilutions at λ_max_ 277 nm using a UV spectrophotometer (UV3600, Shimadzu, Kyoto, Japan), and cumulative percent drug release was calculated. The experiment was performed in triplicate. The release profile of Met from MetNp and MetNp Cap was plotted as time vs. cumulative percent release. In addition, the results obtained were subjected to various release kinetic mathematical models (e.g., zero-order, first-order, Higuchi square root, Korsmeyer–Peppas and, Higuchi kinetics models) to obtain insight on the release mechanism of the drug from the NPs.

### 2.17. Lock Length

Ten distinct capsules were selected for determining lock length. The lock length of each capsule was recorded using Vernier calipers, and the average calculated.

### 2.18. Influence of Storage Conditions on the PS of MetNp

To investigate the influence of storage condition on the PS of the MetNp, the different samples of MetNp were kept in amber-colored glass containers and stored at different temperature conditions: 8 ± 1 °C, 35 ± 2 °C, and 65 ± 2 °C. The samples were analyzed for any significant change in PS after different time periods up to 45 days.

### 2.19. Influence of Storage Conditions on Drug Content

The stability of NPs is a major concern especially during storage of formulations as it is a key constraint, which is considered during large-scale production. The prepared formulations were examined for any change in the drug content after storing samples at two temperature conditions: 8 ± 1 °C and 35 ± 1 °C. Formulations were kept in amber-colored containers for a specified time, and then they were investigated for any change in residual drug content (RDC) after three-time intervals i.e., 15, 30, and 45 days. The samples were measured at 277 nm using a UV-visible spectrophotometer to determine RDC.

### 2.20. Statistical Analysis

All studies were conducted in triplicate (i.e., *n* = 3). After the study, the data were statistically estimated and then expressed in terms of mean ± SD. The data were processed in GraphPad Prism 7.0 software (California, CA, USA) by one-way and two-way ANOVA by using a Turkey–Kramer multiple comparison post-test and Bonferroni post-test, respectively. The results were considered significant at three probability levels of *p* < 0.05, *p* < 0.01, and *p* < 0.001.

## 3. Results and Discussion

During the preliminary investigation of CH-NPs preparation, it was found that variables including the concentration of CH, TPP, and acetic acid; stirring time; and stirring speed affected the development of formulations. From the literature review, it was also found that all these variables affect the preparation of CH-NPs. Due to the large number of variables, it is not beneficial to choose an RSM methodology directly, which increases the experimental runs, time, and cost of experiments. However, these limitations can be overcome by applying 2-level FFD to screen out significant factors that affect CQA, and after, this optimization can be carried out using the RSM method. By taking these goals into consideration, in our experiments, we first applied 2-level FFD to screen out significant factors. In this study, our CQAs were PS and EE.

### 3.1. Testing of Significant/Potential Risk Factors via Applying 2-level FFD

The screening of independent variables was carried out using 2-level FFD. After the experimental analysis, we found that CH concentration, TPP concentration, and stirring speed significantly affect the PS and EE Table 4.

**Figure 3:**
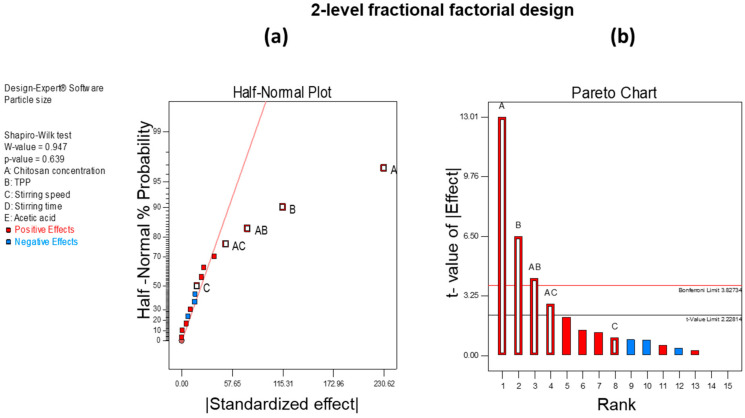
(**a**) Half-normal plot and (**b**) Pareto analysis chart for the screening of independent variables affecting PS.

**Figure 4:**
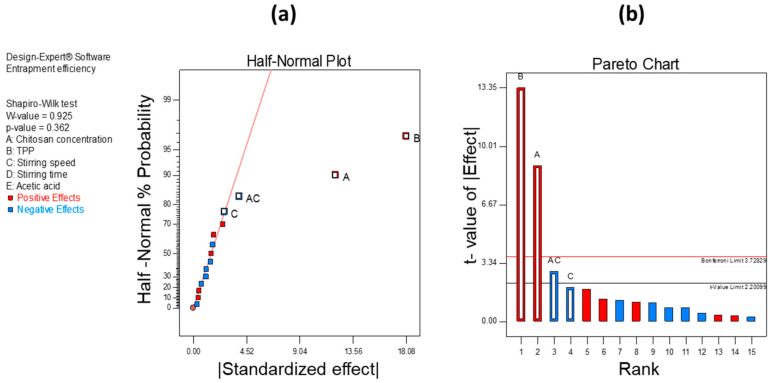
(**a**) Half-normal plot and (**b**) Pareto analysis chart for the screening of independent variables affecting EE.

Figure 3a,b show significant factors through the half-normal plot and Pareto analysis, respectively, that affect PS. From these observations, we found that CH concentration, TPP concentration, and stirring speed significantly affect the PS of the developed formulation. Similarly, in the case of EE, we found that the concentrations of CH and TPP and stirring speed have a significant effect. Figure 4a,b show half-normal plot and Pareto chart analysis, respectively, for EE.

Hence, from the analysis below, we found that CH concentration, TPP concentration, and stirring speed significantly affect both PS and EE of CH-NPs which were further optimized using BBD.

### 3.2. Optimization of CH-NPs for PS and EE

The optimization of CH-NPs was carried out using an RSM methodology, i.e., BBD. In this method, screened independent variables through 2-level FFD were analyzed. The experimental design gave a total of 17 runs for BBD, which were performed and further analyzed for the model. Table 5 shows the experimental results obtained from performing an experiment that describes the influence of selected independent variables (i.e., concentrations of CH and TPP, and stirring speed) on PS and EE.

The model was selected on the basis of maximizing the adjusted R^2^ values and projected R^2^ values. For PS, the quadratic model showed a maximum correlation between adjusted R^2^ values and predicted R^2^ values with minimal difference in their values. The adjusted and predicted R^2^ values obtained for the quadratic model were 0.9448 and 0.8896, respectively, with a difference of less than 0.2. For EE, the quadratic model was the best fit, and adjusted R^2^ values and predicted R^2^ values were 0.9460 and 0.9230, respectively, with a difference lower than 0.2. The model *p*-value should be significant, and the lack of fit value should be nonsignificant. For both the independent variables, we obtained significant values (<0.0001), and lack of fit values were 0.1863 and 0.7758 for PS and EE, respectively, which were nonsignificant. ANOVA was used for statistical analysis. Table 6 and Table 7 give summaries of *p*-value and F value for the model as well as an individual variable for PS and EE.

After ANOVA, a polynomial equation was generated for both of the dependent variables. These reduced polynomial quadratic equations are as follows:

PS = +492.72 + 200.60A + 38.38B + 26.63AB + 80.31A^2^ (10)

EE = +54.28 + 15.60A + 2.21B − 2.79C − 6.59A^2^ (11)

From these equations, we can conclude that a positive coefficient shows a synergistic effect, while a negative sign indicates the antagonistic effect.

**Figure 5:**
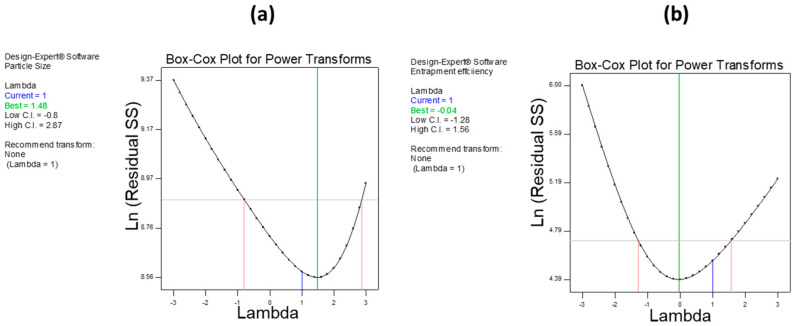
Box–Cox plot for power transformation. (**a**) PS and (**b**) EE. These plots show the lambda value within the limit for both PS and EE, which suggests no power transformation required.

The Box–Cox plot (a diagnostic plot) was used to investigate power transformation for dependent variables. In both responses, we found that no power transformation was required. Figure 5a,b show a Box–Cox plot for PS and EE, respectively.

**Figure 6:**
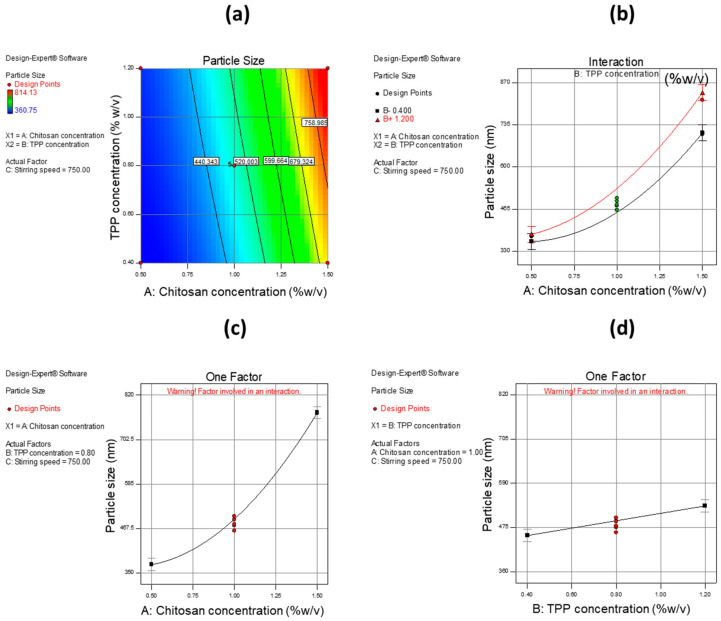
Different interactions and one-factor plot of independent variables as well as their effect on the dependent variable (PS). (**a**) 2-D contour plot showing interaction effect of concentration (% *w*/*v*) of CH and TPP on PS, (**b**) interaction plot showing interaction effect of concentration (% *w*/*v*) of CH and TPP on PS, (**c**) one-factor plot showing the effect of CH concentration (% *w*/*v*) on PS, and (**d**) one-factor plot showing the effect of TPP concentration (% *w*/*v*) on PS.

A 2-D contour plot, interaction plot, and one-factor plots were utilized to estimate the influence of independent variables on dependent variables. CH was found to have a high impact on PS with an increase in the concentration of CH, leading to an increase in PS. The same effect was found with TPP concentration and interaction of CH with TPP, but the effect was much less as compared to CH concentration alone. Figure 6 shows the effect of independent variables on PS using a 2-D contour plot, interaction plot, and one-factor plot. In the case of EE, CH showed a higher impact as compared to other variables i.e., TPP concentration and stirring speed. When CH concentration was increased, an increase in EE was observed. The same phenomenon was also observed with TPP concentration but with a smaller effect. With respect to stirring speed, it was observed that when the stirring rate was increased, a decrease in EE was observed.

Other values including F-value and adequate precision investigated for the model selection and quadratic model were found to fulfil all the criteria for fitting. Table 8 gives information about common parameters and their values. These results suggest that an increased amount of CH allows more of the drug to be captured, while increased stirring speed may cause the entrapped drug to leak out, thus reducing EE.

**Figure 7:**
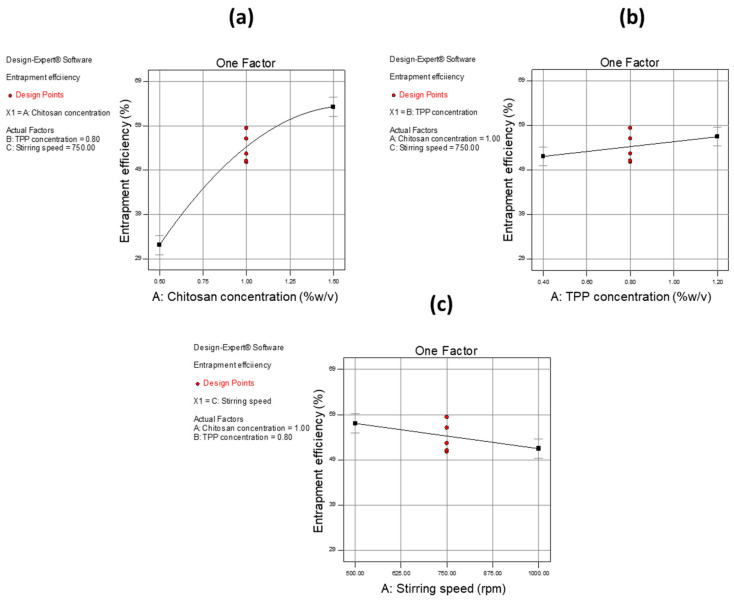
One-factor plots of independent variables as well as their effect on the dependent variable (EE). (**a**) One-factor plot depicting the influence of concentration (% *w*/*v*) of CH on EE, (**b**) one-factor plot depicting the influence of concentration (% *w*/*v*) of TPP on EE, and (**c**) one-factor plot depicting the influence of stirring speed (rpm) on EE.

Figure 7 shows the effect of independent variables on the EE.

### 3.3. Optimization of Design as Well as Validation of the Model

**Figure 8:**
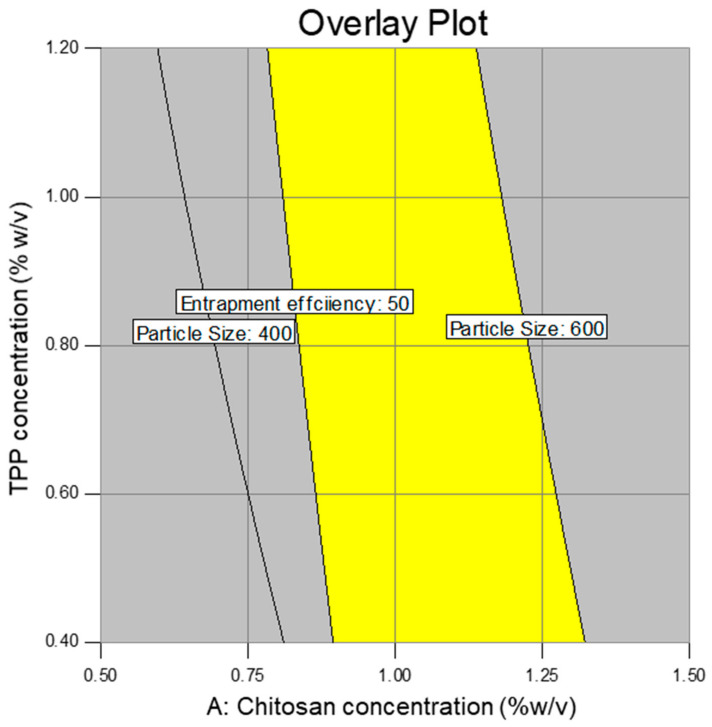
The plot shows the design space (overlay plot) for responses PS and EE.

The optimization of the design, as well as validation of the model, was performed by generating a design space and further performing additional confirmations through the entire design space. Figure 8 shows the generated design space (overlay plot) for responses PS and EE.

After performing the confirmatory experiments, we obtained good agreement among experimental as well as predicted values. The obtained R^2^ values were 0.91 and 0.97 for PS and EE with constraints of 400–600 nm and >50%, respectively—Table 9.

### 3.4. PS and %EE

The optimized MetNp formulations exhibited an average PS of 534.12 ± 1.94 nm. However, after loading Met in the MetNp, the average PS was slightly increased to 558.06 ± 2.52 nm as was expected. Furthermore, drug entrapment analysis of the MetNp formulation revealed good %EE (59.07 ± 2.15%) [36].

### 3.5. Characterization of Blend

The results confirm that all selected parameters (e.g., flow properties) were in the satisfactory range [37,38]. The flow properties of the blend were confirmed by the Hausner ratio and are shown in Table 10. The values obtained for bulk as well as tapped density were observed to be 0.551 and 0.769, respectively. Compressibility index and Hausner’s ratio for powder blend was found to be 25.87% and 1.35, respectively. Eventually, results analysis confirmed good blend flow characteristics of the formulation, which are considered an essential aspect for an ideal blend.

### 3.6. Evaluation of Capsules

The results for capsules are shown in Table 11. They confirm that all the estimating parameters were in the requisite range.

### 3.7. In Vitro Drug Release Study

**Figure 9:**
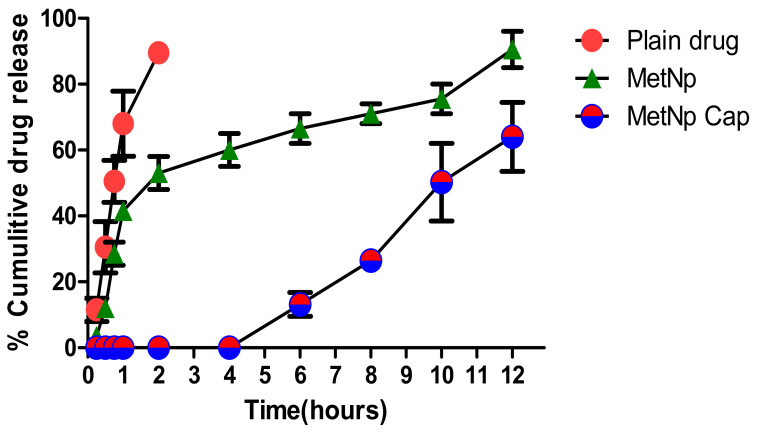
Cumulative percent drug released at different pH of dissolution medium. Results are represented as the mean of 3 determinations on different batches of the same type of dispersion (mean ± SD, *n* = 3). SD indicates standard deviation.

The release of Met was determined from MetNp and MetNp Cap. A burst release (37.02 ± 2.67%) of the drug was seen from MetNp after 1 h, when studied in SGF (pH 1.2), followed by a sustained release pattern of drug with nearly 97.74 ± 3.06% of Met released after 12 h (Figure 9). The drug release pattern from MetNp revealed a biphasic profile, with an early 30–40% burst release of Met in 1–2 h that could be ascribed to both binding of Met at the surface and also embedding of Met superficially in the CH of NPs. Furthermore, uncoated MetNp containing CH and TPP had a significant influence on the release of Met. Both CH and TPP diminished the release of Met from the MetNp. The release of Met was diminished with an increase in the %amount of both CH and TPP. This might be owing to the formation of a strong matrix due to a strong polyelectrostatic interaction amongst the positively charged amino group of the CH and the negatively charged phosphate group of TPP.

The sustained release of Met was due to strong cross-linking amongst CH as well as TPP. This increases the density of the particle owing to the swelling of the CH and thus lessens the permeability of the membrane for the release of Met. In the case of MetNp Cap, no release of Met was observed up to 4 h in SGF (pH 1.2). After 4 h, the release of Met was observed in SIF (pH > 7.0). In SIF (pH > 7.0), the Eudragit S100 coating was dissolved and thus permitted the degradation of the capsule to release the drug. Furthermore, in a sequential drug release study, the formulation was subjected to different simulated GI fluids of a distinct pH environment–Table 12. After 6 h, MetNp Cap released only 15.69 ± 0.82% of drug, and after 12 h, it released nearly 56.84 ± 1.53% of the drug, which suggests its sustained and pH-triggered release pattern of Met through MetNp Cap. Eventually, results indicated a pH-dependent release of drug from the coated capsules.

**Figure 10:**
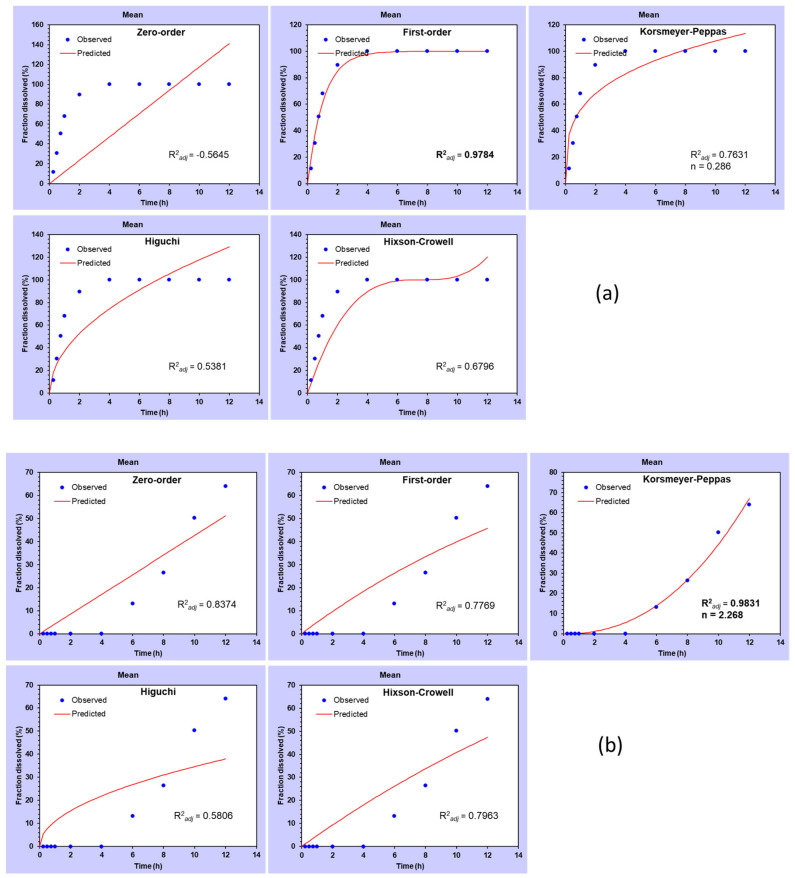

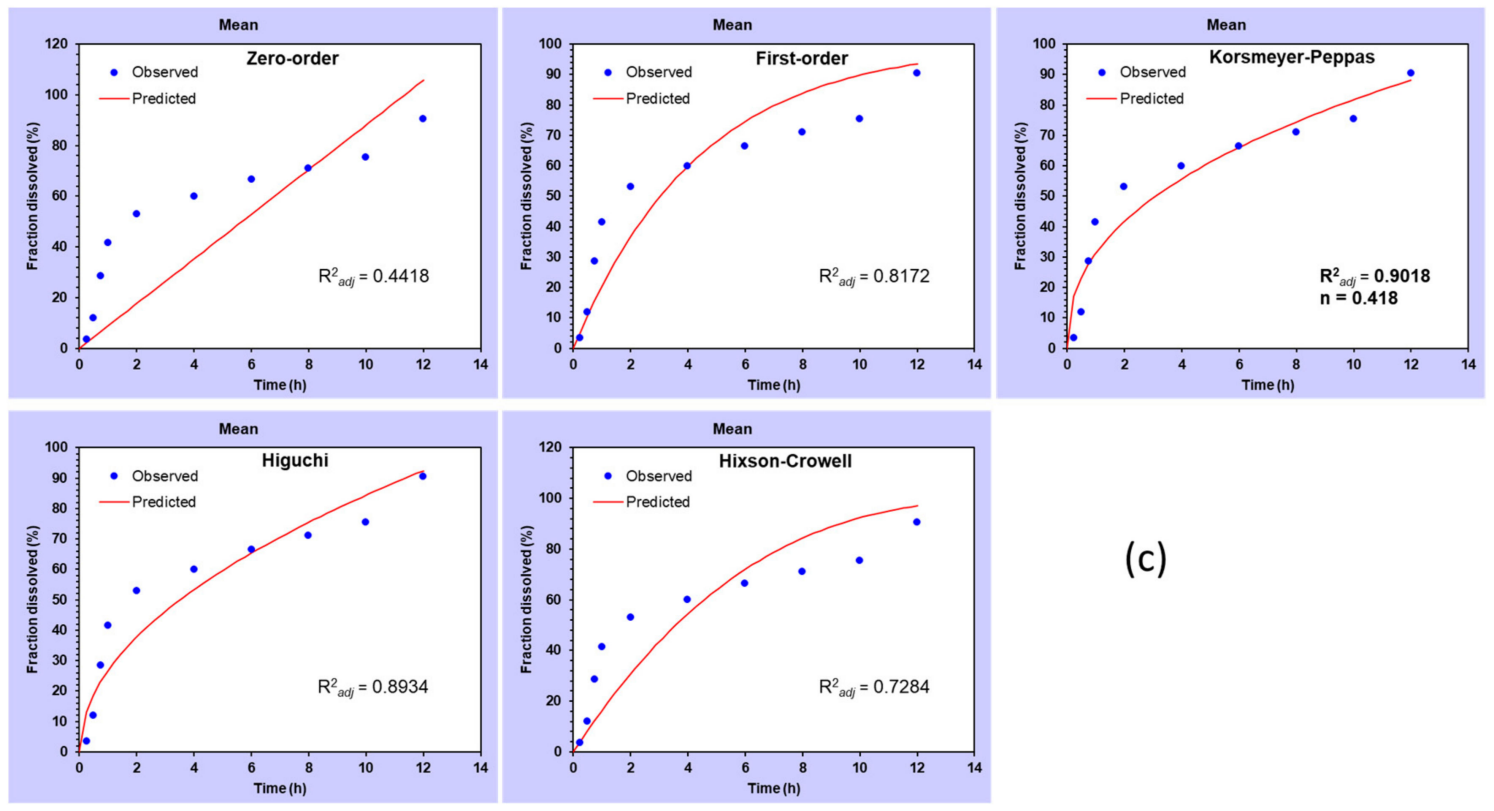
Graphs show different kinetic models for (**a**) plain drug, (**b**) MetNp Cap, and (**c**) MetNp.

Different kinetic models were tested for best-fit after applying data obtained from different formulations (Figure 10). The best-fit kinetic model was selected by comparing their R^2^ adjusted (R^2^*_adj_*) values. The model showing the R^2^*_adj_* value closest to 1 was considered as \best-fitted kinetic model [39]. Drug release data from the plain drug followed zero-order kinetics with the highest R^2^*_adj_* value (R^2^*_adj_* = 0.9784) (Figure 10a). In general, when *n* < 0.45, the release of drug is considered pseudo-Fickian; on the other hand, at *n* = 0.45, the release of drug is referred to as Fickian. The value of n between 0.45 and 0.89 is usually called diffusion as well as swelling-controlled release (i.e., non-Fickian transport). At *n* = 0.89, it is considered zero-order (case II transport), and when the value of n is observed greater than 0.89, then it is termed as super case II transport. MetNp Cap exhibited Korsmeyer–Peppas kinetic as the best-fit model (R^2^*_adj_* = 0.9831, *n* = 2.268) (Figure 10b). The value of n was observed to be ~2.268 (*n* > 0.89), which suggested the super case II transport of Met. The release data from MetNp also showed Korsmeyer–Peppas kinetic as the best-fit model (R^2^*_adj_* = 0.9018, *n* = 0.418) (Figure 10c) [40,41]. While the Korsmeyer–Peppas model was analyzed for *n* value, it was detected close to 0.418 (*n* < 0.45), which confirmed that the MetNp formulation followed pseudo-Fickian transport.

### 3.8. RDC

The optimized MetNp were kept at 8.0 ± 1 and 35.0 ± 1 °C, and the RDC of the MetNp was measured at different time intervals: 15, 30, and 45 days. The percent RDC of the selected MetNp is presented in Table 12. It was noticed that the formulation stored at 8.0 ± 1 °C was quite stable, as much less drug (0.37%) was deprecated on storage for 45 days, while product stored at 35.0 ± 1 °C was quite unstable compared to at 8.0 ± 1 °C. About 2.15% of the drug degraded upon storing at 35.0 ± 1 °C for 45 days. Eventually, these results suggested a slight decrease in the %EE of the Met after 45 days; however, it was within the consented limit [36].

### 3.9. Long-Term Stability of MetNp

**Figure 11:**
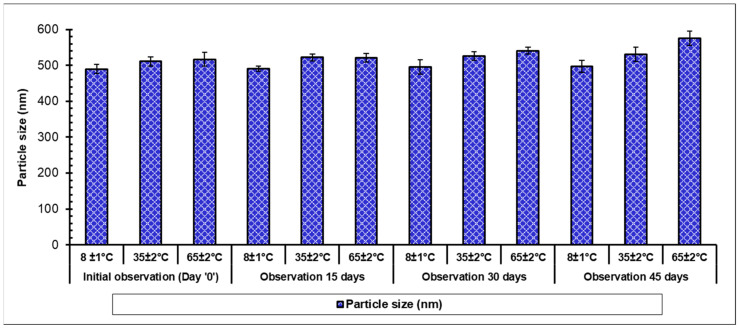
Influence of storage conditions on PS of MetNp.

The stability of the formulations can be defined as the ability of the product to retain its definite amount of drug for a scheduled time, which is also termed as the shelf-life of the formulation. It is the aptitude of a product packed in a particular container to endure the chemical, microbiological, physical, therapeutic, and toxicological requirements and is a major prerequisite for its successful clinical translation. A formulation can be considered stable if it preserves not only morphology along with integrity but also retains several features including the nature, its content, and release profile of the active molecules. In most of the stability investigations, the foremost importance is being shown against accelerated stability related issues; however, stability investigations of aged formulations have depicted more pharmaceutical significance. Stability studies were carried out as per International Council for Harmonization (ICH) guidelines [42] using optimized MetNp formulations, which were stored as a powder for a period of 45 days at 8.0 ± 1 °C, 35.0 ± 2 °C, and at 65 ± 2 °C and observed at 15-day intervals (Figure 11). PS was slightly increased in storage during the stability study, which might be due to the attraction and agglomeration of small NPs leading to an increase in the size of NPs. Finally, studies revealed the stability of the formulation.

## 4. Conclusions

The application of the QbD approach and risk assessment method demonstrated its potential by selecting DOE and statistical analysis. In this investigation, these approaches helped in the development of MetNp using CH and TPP. Furthermore, QbD was successfully applied to determine the quality attributes of MetNp. FFD was used by using five independent variables viz., concentrations of CH, TPP, and acetic acid as material attributes as well as stirring speed and stirring time as process parameters, whereby their effect on two dependent variables viz., PS, and %EE, was studied. MetNp were prepared using a modified ionic gelation method based on an optimized formula obtained from the above QbD design study. PS and %EE studies revealed the formation of MetNp with 558.06 ± 2.52 nm and 59.07 ± 2.15%, respectively. Furthermore, a drug release study using various simulated GI fluids suggested a pH-triggered (pH > 7.0) and sustained release profile of Met from Eudragit S100 enteric-coated MetNp cap. Stability investigation of formulations confirmed good stability with respect to their PS and RDC at different temperature conditions. In conclusion, QbD methodology was fruitfully applied in the development of MetNp and enteric-coated MetNp cap depicting their potential to release Met through MetNp cap only in the colon region and thus making them suitable for the treatment of amoebiasis in the colon.

## Figures and Tables

**Table 1:** 2-level FFD to determine the influence of various critical material attributes (CMA) and critical process parameters (CPP) on particle size (PS) and entrapment efficiency (EE) of NPs.

Independent Variables	Units	Levels	
		Low (−1)	High (+1)
CH concentration	(% *w*/*v*)	0.5	1.5
TPP concentration	(% *w*/*v*)	0.4	1.2
Stirring speed	(rpm)	500	1000
Stirring time	(min)	30	90
Acetic acid	(% *v*/*v*)	1	1.5

**Table 2:** High, medium and low levels for BBD to determine the influence of concentration of CH, TPP, as well as stirring speed on PS and EE of CH-NPs.

Variables	Units	Levels		
		High (+1)	Medium (0)	Low (−1)
CH concentration	% *w*/*v*	0.5	1	1.5
TPP concentration	% *v*/*v*	0.4	0.8	1.2
Stirring speed	rpm	500	750	1000

**Table 3:** Time schedule of various simulated gastrointestinal (GI) fluids for drug release (in vitro) investigations.

Type of GI Fluid	Time (h)
Inside SGF (pH 1.2)	0–2
Inside SGIF (pH 4.5)	3–4
Inside SIF (pH 7.4)	5–8
Inside SCF (pH 7.0)	9–10

**Table 4:** Design of experiments (DOE) results (2-level FFD) to study the effect of different CMA and CPP on PS and EE.

Formulation Batch	Independent Variables					Dependent Variables	
	CH Concentration (% *w*/*v*)	TPP Concentration (% *w*/*v*)	Stirring Speed (rpm)	Stirring Time (min)	Acetic Acid (% *v*/*v*)	PS (nm)	EE (%)
F1	1.5	1.2	500	90	1	705.91	68.12
F2	1.5	0.4	1000	30	1.5	540.8	38.18
F3	0.5	1.2	500	90	1.5	416.3	45.47
F4	1.5	1.2	500	30	1.5	630.75	65.02
F5	0.5	0.4	500	90	1	419.01	30.23
F6	0.5	0.4	500	30	1.5	400.64	35.75
F7	0.5	1.2	500	30	1	430.01	47.37
F8	0.5	0.4	1000	90	1.5	390.30	29.42
F9	1.5	0.4	500	30	1	520.95	45.81
F10	1.5	1.2	1000	30	1	775.16	59.30
F11	0.5	1.2	1000	30	1.5	425.06	49.09
F12	1.5	0.4	1000	90	1	550.83	40.70
F13	0.5	1.2	1000	90	1	409.37	50.50
F14	1.5	1.2	1000	90	1.5	790.66	58.36
F15	0.5	0.4	1000	30	1	309.80	34.83
F16	1.5	0.4	500	90	1.5	530.35	43.71

**Table 5:** Experimental results of DOE (BBD) to investigate the influence the concentrations of CH and TPP, and stirring speed on PS analysis and EE of NPs.

Formulation Batch	Independent Variables			Dependent Variables	
	CH Concentration (% *w*/*v*)	TPP Concentration (% *v*/*v*)	Stirring Speed (rpm)	PS (nm)	EE (%)
B1	1	0.8	750	500.10	58.44
B2	1.5	0.8	500	795.94	65.42
B3	0.5	0.8	500	370.97	32.66
B4	1.5	0.4	750	703.97	61.21
B5	1	0.8	750	490.75	56.08
B6	0.5	0.4	750	377.19	29.73
B7	1	0.8	750	461.85	51.08
B8	1	0.4	500	436.39	59.15
B9	0.5	1.2	750	380.83	35.49
B10	1	0.4	1000	480.76	48.18
B11	1	1.2	1000	549.32	55.10
B12	1	0.8	750	475.20	50.79
B13	1.5	0.8	1000	780.49	58.17
B14	1	1.2	500	561.09	57.02
B15	1	0.8	750	479.01	52.67
B16	0.5	0.8	1000	360.75	30.50
B17	1.5	1.2	750	814.13	68.37

**Table 6:** ANOVA table of a reduced quadratic model for PS.

Source	Sum of Square	df	Mean Square	F Value	*p*-Value	Comments
Model	3.639 × 10^5^	4	90965.22	204.14	<0.0001	Significant
A-CH concentration (% *w*/*v*)	3.219 × 10^5^	1	3.219 × 10^5^	722.42	<0.0001	-
B-TPP concentration (% *w*/*v*)	11785.73	1	11785.73	26.45	0.0002	-
AB	2836.63	1	2836.63	6.37	0.0268	-
A^2^	27319.67	1	27319.67	61.31	<0.0001	-
Residual	5347.32	12	445.61	-	-	-
Lack of Fit	4483.8	8	560.48	2.60	0.1863	not significant
Pure Error	863.47	4	215.87	-	-	-
Cor Total	3.692 × 10^5^	16	-	-	-	-

**Table 7:** ANOVA table of a reduced quadratic model for EE.

Source	Sum of Square	df	Mean Square	F Value	*p*-Value	Comments
Model	2231.59	4	557.90	71.10	<0.0001	significant
A-CH concentration (% *w*/*v*)	1946.57	1	1946.57	248.06	<0.0001	-
B-TPP concentration (% *w*/*v*)	39.21	1	39.21	5.00	0.0452	-
Stirring speed (rpm)	62.16	1	62.16	7.92	0.0156	-
A^2^	183.66	1	183.66	23.40	0.0004	-
Residual	94.16	12	7.85	-	-	-
Lack of Fit	49.70	8	6.21	0.56	0.7758	not significant
Pure Error	44.46	4	11.12	-	-	-
Cor Total	2325.76	16	-	-	-	-

**Table 8:** Statistical data values for dependent variables.

Parameters	Values for Dependent Variables	
	PS	EE
Model F value	204.14	71.10
Model *p*-value	<0.0001	<0.0001
Lack of fit *p*-value	0.1863	0.7758
R^2^ unadjusted value	0.9855	0.9595
R^2^ adjusted value	0.9807	0.9460
R^2^ predicted value	0.9636	0.9230
Adequate precision	41.750	24.205

**Table 9:** Quality Target Product Profile (QTPP) for QbD assisted formulation of MetNp.

Parametric Components	Target Profile
Dosage formulation	NPs for target-specific delivery
Drug release profile	Sustained release for prolonged effect of Met
PS	400–600 nm
EE	>50%
Drug release	>36 h

**Table 10:** Blend parameters with observed values.

Blend Parameters	Observed Value
Bulk density (gm/cc)	0.551
Tapped density (gm/cc)	0.769
Compressibility index (%)	25.87
Hausner’s ratio	1.35
Blend flow characteristics	Good

**Table 11:** Capsule parameters with observed values.

Capsule Parameters	Observed Values
Selected capsule size	#4
Capsule empty weight	38 ± 3 mg
Capsule fill weight	120 mg
Lock length	14.1–14.15 mm

**Table 12:** Influence of storage on residual drug content (RDC) of MetNp on storage at different temperatures.

Initial Drug Concentrations (%)	% RDC					
	8.0 ± 1 °C			35.0 ± 1 °C		
	Days					
	15th	30th	45th	15th	30th	45th
100	99.86	99.75	99.63	98.47	98.15	97.85

## Abbreviations

%EE	Percent entrapment efficiency
ANOVA	One-way analysis of variance
BBD	Box–Behnken design
CH	Chitosan
CH-NPs	Chitosan NPs
CMA	Critical material attributes
CPP	Critical process parameters
CQA	Critical quality attributes
DOE	Design of experiments
E.	Entamoeba
FFD	Fractional factorial design
GI	Gastrointestinal
ICH	International Council for Harmonization
Met	Metronidazole
MetNp	Met containing NPs
MetNp Cap	Eudragit coated capsule containing MetNp
MTP1	Metal tolerance proteins-1
NPs	Nanoparticles
PNP	Purine nucleoside phosphorylase
PS	Particle size
QbD	Quality by design
QTPP	Quality target product profile
R^2^adj	Adjusted coefficient of determination
RDC	Residual drug content
RSM	Response surface methodology
S_c_	Surface area of the uncoated capsule
SCF	Simulated colonic fluid
SGF	Simulated GI fluid
SGIF	Simulated gastric and intestinal fluid
SIF	Simulated intestinal fluid
SOD	Superoxide dismutase
TPP	Tripolyphosphate
Trx	Thioredoxin
TrxR	Thioredoxin reductase
ρ_o_	Bulk density
ρ_t_	Tapped density
W_c_	Weight of uncoated capsule
W_ec_	Weight of enteric-coated capsule
